# Hippocampal gray matter volume in the long-term course after transient global amnesia

**DOI:** 10.1016/j.nicl.2021.102586

**Published:** 2021-02-12

**Authors:** Mandy Pirlich, Cathleen Höfer, Christopher M. Weise, Anika Stockert, Angelika Thöne-Otto, Alexander Garthe, Stefan Schob, Joseph Classen, Karl-Titus Hoffmann, Dorothee Saur

**Affiliations:** a Department of Neurology, University of Halle Medical Center, Halle, Germany; b Department of Neurology (M.P., C.H., C.M.W., A.S., J.C., D.S.), Department of Neuroradiology (S.S., K.T.H.) and Department of Cognitive Neurology (A.T.O.), University of Leipzig Medical Center, Leipzig, Germany, German Center for Neurodegenerative Diseases, Dresden (A.G.), Germany

**Keywords:** Memory deficits, Water maze, Gray matter volume, Voxel-based morphometry

## Abstract

**Objective:**

In this retrospective, cross-sectional study we aimed to examine long-term memory deficits and gray matter volumes (GMV) in the hippocampus after transient global amnesia (TGA).

**Methods:**

20 patients with a history of TGA (TGA+, mean 6.5 years after TGA) and 20 age-matched healthy controls (TGA-) underwent neurocognitive assessment (i.e. Mini-Mental State Examination (MMSE), visuospatial, verbal and episodic autobiographical memory and visuospatial learning/navigation [“human water maze”]) in combination with structural cerebral MRI. Voxel-based morphometry (VBM) was used to detect GMV in the hippocampus in TGA+ versus TGA-.

**Results:**

Besides slight differences in MMSE and visuo-spatial learning/navigation measured with a human water maze in TGA+ vs. TGA-, no other tests of visuo-spatial, verbal and autobiographical long-term memory differed between groups. VBM analyses yielded a statistically significant difference in bilateral hippocampal GMV with TGA+ compared to TGA- showing greater GMV in a region corresponding to bilateral CA1. However, none of the hippocampus-dependent cognitive measures correlated with hippocampal GMV.

**Conclusion:**

In the long-term course after TGA, only subtle neurocognitive deficits without microstructural damage of the hippocampus could be detected. Greater GMV in bilateral hippocampus in TGA+ vs. TGA- may indicate that TGA triggers hippocampal GMV increase rather than atrophy.

## Highlights

• No substantial hippocampus-dependent memory deficits in the long-term course after transient global amnesia.
• Greater hippocampal gray matter volume in patients with transient global amnesia compared to healthy controls in the long-term course.
• Transient global amnesia might trigger neuronal and/or non-neuronal mechanisms in the hippocampus resulting in an increase of grey matter rather than atrophy.

## Graphical abstract

**Figure:**
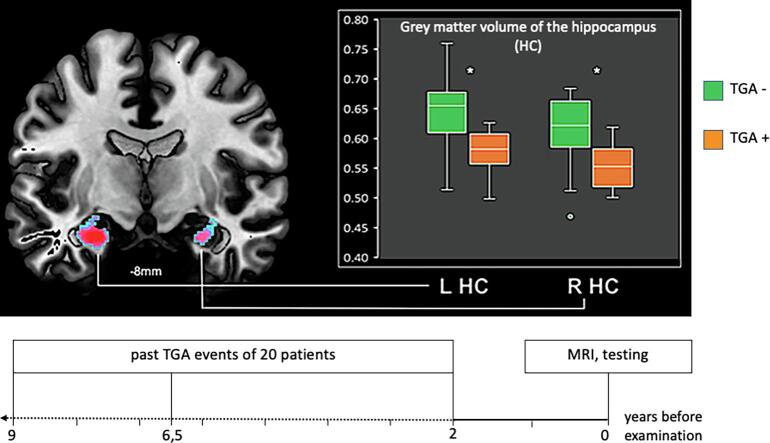

## 1 Introduction

Transient global amnesia (TGA) is a temporary isolated impairment of episodic memory resulting in anterograde and – to a lesser degree – retrograde amnesia. During acute TGA, transfer of multimodal information into episodic memory is disturbed while general cognitive functions remain intact (Qinette et al., 2003). Although these deficits of episodic memory are considered temporary, it has been hypothesized that subclinical memory impairments could persist over time (Bartsch et al., 2006). Supporting studies found subtle impairments in verbal and nonverbal memory days (Kessler et al., 2001), months (Mazzuchi et al., 1980) or even a year (Borroni et al., 2004) after TGA. Recently, prolonged allocentric navigation deficits were demonstrated three months after TGA (Schöberl et al., 2019). In contrast, a *meta*-analysis of 25 studies reported complete recovery of cognitive functions 5 days to 6 months after TGA (Jäger et al., 2009).

Diffusion-weighted imaging (DWI) frequently reveals focal hyperintense lesions in the lateral hippocampus corresponding to the CA1 region. The CA1 region is known to be vulnerable to cytotoxic noxae, and lesions of CA1 neurons can be considered the structural correlate of the amnestic deficit (Bartsch et al., 2015). Although the exact mechanism remains unclear, these focal lesions and possible subsequent alterations of hippocampal structure may be critical to the pathogenesis and long-term outcome of TGA.

In this retrospective, cross-sectional study, we combined neurocognitive testing (verbal/visuospatial learning and memory) with voxel-based-morphometry (VBM) to examine long-term memory deficits and brain structural alterations after TGA. We compared patients with a history of TGA that occurred at least 2 years before scanning (TGA+) with healthy controls (TGA-). We hypothesized that TGA+ compared to TGA- would exhibit reduced hippocampal gray matter volume (GMV) associated with long-term impairments in episodic verbal and visuospatial memory following TGA.

## 2 Methods

### 2.1 Patients and controls

*Patients:* To identify patients with a past episode of TGA, we reviewed the diagnosis of patients who were admitted to the Department of Neurology, University of Leipzig Medical Center, from 2005 to 2012. In this time period, 224 patients received the diagnosis of TGA according to the ICD classification system (i.e. ICD codes G45.42 or G45.43). The medical records of these patients were then carefully reviewed with regard to the following criteria: Inclusion criteria: (i) Clinical diagnosis of TGA according to the Hodges and Warlow criteria (Hodges & Warlow, 1990), i.e. the attack must be witnessed and information available from a capable observer who was present for most of the attack, clear-cut anterograde amnesia during the attack, cognitive impairment limited to amnesia without clouding of consciousness or loss of personal identity, no accompanying focal neurologic symptoms during the attack and no significant neurologic signs afterward, absence of epileptic features and resolution of the attack within 24 h, (ii) German native speakers and (iii) ability to give written informed consent. The presence of typical DWI lesions was not an inclusion criterion. Exclusion criteria: (i) Fulfilment of the Hodges and Warlow criteria could not be inferred with certainty from the documented history, clinical, neurological and neurocognitive examination, (ii) structural brain lesions documented on cranial MRI at the time of the TGA, (iii) poor condition of general health, (iv) contraindications for MRI scanning and (v) factors confounding the neurocognitive testing such as alcohol abuse, dyslexia, visual and hearing deficits. 31 patients who were contactable met these inclusion and exclusion criteria. From this cohort, we randomly selected 20 patients who agreed to participate in the study. *Healthy Controls:* 20 controls were German native speakers without a history of neurological or psychiatric illness that matched the TGA cohort in age, sex and educational background (n = 7 spouses). Patients and healthy controls were invited to complete a neurocognitive assessment followed by a cranial MRI scan. All participants received a monetary reimbursement of 25 Euro. The study was approved by the Ethics Committee of the Medical Faculty at the University of Leipzig (095–14-10032014). All subjects gave their written informed consent to participate in the study.

### 2.2 Data acquisition

*Neurocognitive assessment.* Testing included the German versions of the MMSE (Folstein et al., 1975), the path subtest of the Visual and Verbal Memory Test (Visueller und Verbaler Merkfähigkeitstest, VVM-path), (Schellig and Schaechtele, 2009) and the short version of the California Verbal Learning Test (CVLT), (Niemann et al. 2008). In addition, we used the Dresden Spatial Navigation Task, (Woost et al. 2018), which represents a human adaptation of the Morris Water Maze (huWMZ). With these tests, we aimed to assess (i) verbal (CVLT) and (ii) visuospatial (VVM-path) memory as well as (iii) visuospatial learning/navigation (huWMZ). Based on previous studies demonstrating impairment of episodic autobiographical memory in patients with TGA (Bartsch et al. 2011), we additionally used the Autobiographical and Semantic Memory Interview (part 1), to assess episodic memories of the most recent five-year-period (Schmidtke & Vollmer-Schmolck, 1999). In the following, tests and dependent variables are described in more detail.

*VVM-path.* The VVM-path involves recalling and drawing a path into a city map after a memorizing time of two minutes. Recall is assessed immediately after memorization as well as after a 20 min delay. The original path is divided into several units, credits are given for each unit correctly recalled (maximum score 31). The resulting dependent variables were VVM-path 1 (immediate recall) and VVM-path 2 (delayed recall).

*Short-version of the CVLT.* The CVLT is a 9-item list of three categories, which is presented 4 times. It is a measure of verbal learning and memory, which assesses free recall and recognition. Our variables of interest were verbal learning (immediate free recall, i.e. total number of items recalled after each of the 4 runs, maximum learning score 36) and verbal memory (delayed free recall of the wordlist after 10 min, maximum score 9).

*Maze Training with Dresden Spatial Navigation Task.* To familiarize participants, spatial training started with 2 practice trials to introduce the game pad. In the following 12 trials, participants were asked to find a hidden platform in an identical position from 3 different starting points. Mazes were visualized through a first-person perspective on a standard computer screen. To generate an allocentric spatial perspective, mazes were surrounded by concentrically placed objects of a swimming pool ambience. Participants were given 60 s for solving the navigation task. As soon as participants found the hidden platform within the time limit, or immediately after the 60 s had passed without finding the hidden platform and being towed to it, the next trial was initiated. The two practice trials had a fixed length and no hidden goal to be found. Allocentric visuospatial learning and navigation were operationalized by two variables of interest: (i) path length (arbitrary units) and (ii) latencies to successful completion of navigation toward the platform (seconds). A value of 60 sec was assigned to trials in which the platform was not reached. Lower values of either variable indicated better navigation performance.

*Episodic Autobiographical Memory Score.* Participants were asked about different events in the past five years referring to topics like travelling, festivals, work and living (maximum score 38).

*MRI.* Anatomical T1-weighted, high-resolution (0.7-mm isotropic voxels) images (MPRAGE) were acquired on a 3 T Scanner (Siemens MAGNETOM Tim Trio) with a 24-channel head coil using the following parameters: repetition time (TR) = 2500 ms, echo time (TE) = 2.45 ms, inversion time (TI) = 1100 ms, flip angle (FA) = 7°. The total acquisition time (TA) for the MPRAGE was 5:09 min. Additional sequences (DWI, T2) were used for visual inspection (see below).

### 2.3 Data analysis

*Behavioral data.* For statistical analyses of neurocognitive test data, we used IBM SPSS Statistics v20.0. Comparisons between independent groups were performed with two-sample t-tests after testing for normal distribution (Kolmogorov-Smirnov) and with chi-square tests. The threshold of significance was set to p < 0.05 (two-tailed). In addition, Pearson partial correlation was used for post-hoc correlational analyses of extracted hippocampal GM values (i.e. residuals of the respective cluster averages after correction for total intracranial voume (TIV)) and cognitive measures.

*MRI:* To control for bias of cognitive impairment due to vascular or neurodegenerative disease, qualitative visual inspection of the MRI scans was done by a board-certified neuroradiologist (K.T.H.) blinded for TGA status. This included quantification of periventricular and deep white matter hyperintensities according to the Fazekas-scale based on T2-sequences (Fazekas et al., 1987) and medial temporal lobe atrophy according to the Scheltens-scale based on MPRAGE (Scheltens et al., 1992). In addition, the hippocampus was carefully inspected for macrostructural abnormalities.

*Voxel-based morphometry*: VBM of T1-weighted MPRAGE images was done using Statistical Parametric Mapping package (SPM12, Wellcome Department of Imaging Neuroscience, London, UK; www.fil.ion.ucl.ac.uk/spm) and the CAT12 Toolbox (www.dbm.neuro.uni-jena.de/cat/). In a first step, structural images were segmented into gray matter (GM), white matter and cerebrospinal fluid. The resulting GM partial volume images were then aligned to standard MNI space using the DARTEL template. Next, the modulated segmented images were smoothed with a full-width-half-maximum isotropic Gaussian kernel with a sigma of 8 mm. Within SPM, two-sample t-tests were applied to investigate brain structural differences in TGA+ vs. TGA-. For all VBM analyses, TIV was included as additional covariate. Since we had a strong a-priori hypothesis regarding the hippocampus, we performed region of interest (ROI)-based analyses in addition to whole-brain analyses. For ROI-based analyses we made use of a standard hippocampus mask (bilateral) generated with the WFU-PickAtlas toolbox (www.fmri.wfubmc.edu). Statistical significance was determined using the Threshold-Free Cluster Enhancement (TFCE) toolbox (dbm.neuro.uni-jena.de), which does not require the arbitrary definition of voxel-wise or cluster thresholds and provides robust non-parametric, permutation-based statistics (Smith and Nichols, 2009). All TFCE-based analyses (both whole-brain and ROI analyses) were performed with 5000 permutations and were considered significant at p < 0.05 FWE corrected. MRIcron software (www.people.cas.sc.edu/rorden/mricron) was used for illustration off imaging data and the SPM Anatomy Toolbox (www.fz-juelich.de/inm/inm-1/DE/Forschung/-docs/SPMAnatomyToolbox) was used for additional subfield identification within the hippocampal ROI.

## 3 Results

### 3.1 Study sample

Characteristics of the patients (TGA+) and healthy controls (TGA-) are presented in Table 1. Mean time between the TGA event and scanning for TGA + was 6.5 years with a range from 2 years to 9 years. As revealed by two-sample t-tests and chi-square tests, groups did not significantly differ regarding age, sex and level of education (see Table 1).

**Table 1:** Overview of the study sample, neurocognitive scores and MRI findings.

Characteristics	TGA (+)	TGA (−)	p
	n = 20	n = 20	
Age (years), M (SD)	69.9 (5.7)	68.2 (6.4)	*ns^1^*
Women (%)	70	65	*ns^2^*
Higher education entrance qualification (%)	30	35	*ns^2^*
Years between TGA and assessment, M (IR)	6.45 (7)		
MMSE (0–30), M (SD)	27.90 (1.86)	28.90 (0.85)	*0.038^1^*
MMSE, delayed recall (0–3), M (SD)	1.60 (1.95)	1.95 (0.88)	*ns^1^*
VVM path 1, immediate recall (0–31), M (SD)	14.45 (4.92)	17.05 (6.21)	*ns^1^*
VVM path 2, delayed recall (0–31), M (SD)	13.65 (5.82)	14.40 (6.41)	*ns^1^*
CVLT, immediate free recall (0–36), M (SD)	27.60 (4.68)	26.65 (4.09)	*ns^1^*
CVLT, delayed free recall (0–9), M (SD)	6.55 (1.95)	6.95 (1.31)	*ns^1^*
Episodic autobiographical memory (0–38), M (SD)	32.55 (5.25)	32.95 (3.69)	*ns^1^*
Human Water Maze, path length measure	F*_2,12_* = 5.04	*0.0323*	
Human Water Maze, latency measure	F*_2,12_* = 1.73	*ns^3^*	
White matter hyperintensities, Median (IR)	1 (1)	1 (1)	*ns^4^*
(Fazekas-scale 0–3)			
Left Medial temporal lobe atrophy, Median (IR)	1 (0)	1 (0)	*ns^4^*
Right Medial temporal lobe atrophy, Median (IR)	1 (0)	1 (0)	*ns^4^*
(Scheltens-scale 0–4)			

Abbreviations: TGA (+) = patients with a history of TGA; TGA (−) = healthy controls; M = mean; SD = standard deviation of the mean; IR = interquartile range; ns = not significant; MMSE = Mini Mental State Examination, VVM = Visual and Verbal Memory Test; CVLT = California Verbal Learning Test.

With exception of the MMSE and path length measure (Human Water Maze), groups did not differ concerning the presented characteristics as revealed by two-sample t-tests^1^, chi-square tests^2^, variance analysis^3^ and Mann-Whitney-U-tests^4^.

### 3.2 Neurocognitive scores

*Mini Mental State Examination (MMSE)*. As shown in Table 1, we found a small difference between the groups for global cognitive functions (MMSE: TGA + 27.90 ± 1.86; TGA- 28.90 ± 0.85; *t* [38] = 2.185; p = 0.038). In a subanalysis, in which only the retrieval task (delayed recall of 3 words) was considered, no significant difference was noted (MMSE-delayed recall: TGA + 1.60 ± 1.09; TGA- 1.95 ± 0.88; t [38] = 1.110; p = 0.274). After reviewing the descriptive data, we removed an outlier in TGA+ (MMSE Score 22 points) which lowered the result to trend-level significance only (MMSE: TGA + 28.21 ± 1.273; TGA- 28.90 ± 0.85; t [37] = 1.997; p = 0.057). There was no association between the MMSE score and demographic variables (age, education) or years since TGA episodes. *VVM-path.* There was no difference between groups (TGA + vs. TGA-) regarding the variables VVM-path 1 (immediate recall) and VVM-path 2 (delayed recall) (F*_1,38_* = 0.062; *p* = 0.805). *California Verbal Learning Test (CVLT).* No group difference was found either for verbal memory (CVLT-immediate free recall: TGA + 27.60 ± 4.68; TGA- 26.65 ± 4.09; *t* [38] = -0.683; *p* = 0.499) or CVLT- delayed free recall: TGA + 6.55 ± 1.95; TGA- 6.95 ± 1.31; *t* [38] = 0.758; *p* = 0.454). *Episodic Autobiographical Memory Score.* There were no differences among groups for episodic autobiographical memory (TGA + 32.55 ± 5.25; TGA- 32.95 ± 3.69; *t* [38] = 0.278; *p* = 0.782).

**Fig. 1:**
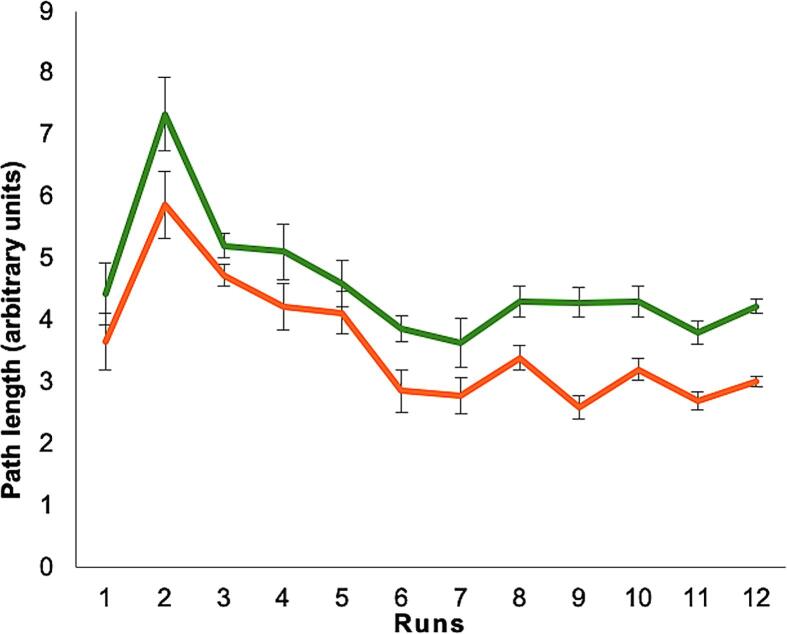
Human Water Maze (huWMZ) performance. Path lengths (in data units) in the huWMZ for TGA+ (green line) and TGA- (orange line). TGA patients revealed a significant longer path length compared with controls. (For interpretation of the references to colour in this figure legend, the reader is referred to the web version of this article.)

*Human Water Maze performance.* In the two practice trials to introduce the game pad, there were no group differences between TGA + and TGA- (latencies: TGA + 59.95 ± 0.22 sec; TGA- 59.94 ± 0.23 sec; *t* [36] = 0.073; *p* = 0.942; path length: TGA + 3.60 ± 4.13, TGA- 2.41 ± 2.07; *t* [36] = 1.13; *p* = 0.265). During the 12 acquisition trials (spatial acquisition), patients and controls did not differ in the latency measure (F*_2,12_* = 1.73; *p* = 1.96). However, differences were detected in the path length measure (F*_2,12_* = 5.04; *p* = 0.032). That is, TGA patients needed longer paths to find the hidden platforms (Fig. 1).

### 3.3 MRI evaluation

Qualitative visual inspection of the MRI scans revealed relevant macrostructural abnormalities in terms of hippocampal cysts and gliosis in two participants, one with and one without TGA. According to the quantification of white matter hyperintensities and medial temporal lobe atrophy, scoring did not differ significantly between groups. With respect to the age of our cohorts, overall microvascular damage and hippocampal atrophy was low in both groups (Table 1).

### 3.4 VBM analysis

**Fig. 2:**
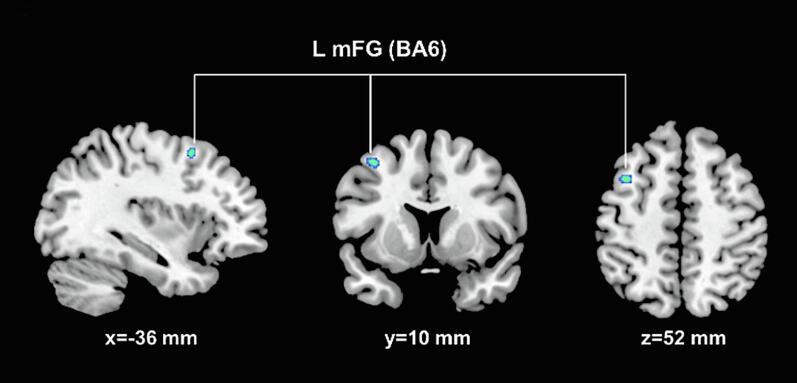
Lower regional GMV in TGA + vs. TGA-. Significant (p < 0.05 FEW) lower regional GMV in TGA + vs. TGA- in the sagittal, coronal and axial plane (from left to right) with the corresponding location in MNI space. L mFG, Left middle frontal gyrus; BA, Brodmann Area-36.

**Fig. 3:**
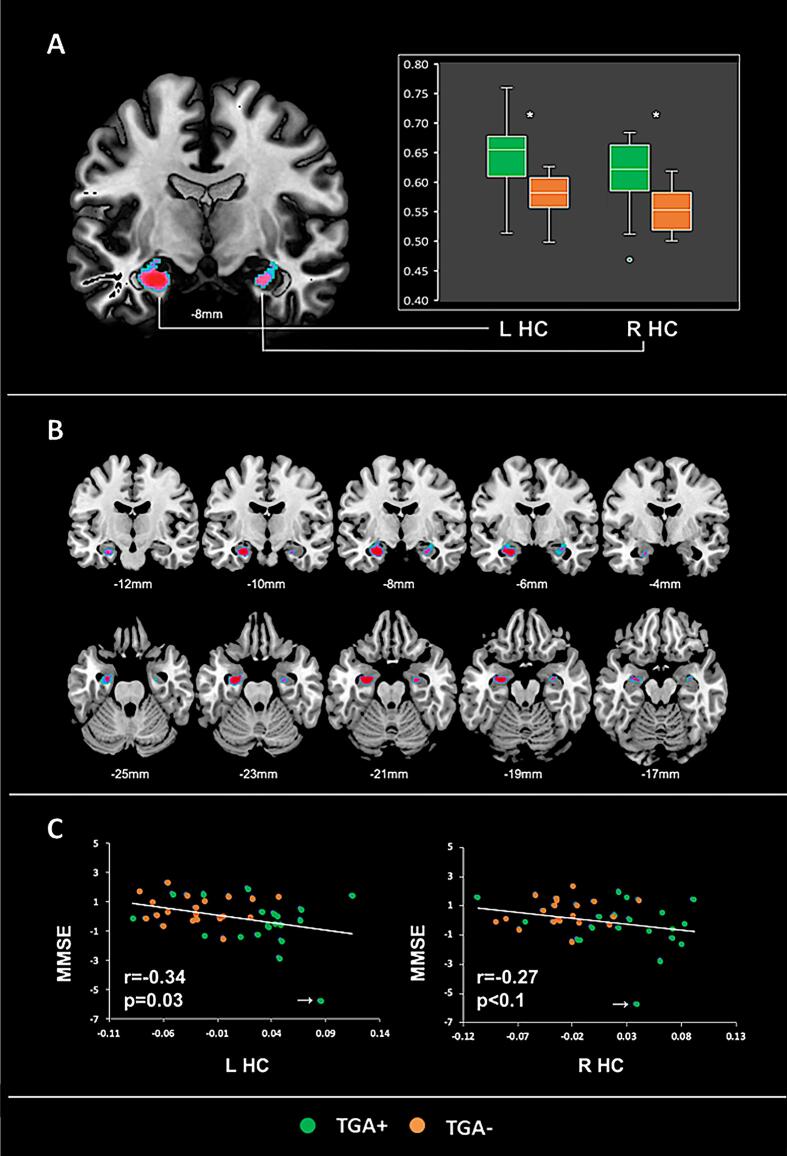
Greater regional GMV (VBM) in TGA + vs. TGA-. (A) ROI-based VBM analyses show greater increased hippocampal GMV in TGA + vs. TGA- with corresponding box blots of extracted raw GM values. (B) Coronal (top row) and axial (bottom row) sections display the full extent of hippocampal GMV changes (p < 0.05 FEW_SVC_) in TGA + vs. TGA-. Coordinates indicate the corresponding location on the y-axis (top row) and z-axis (bottom row), respectively. (C) Scatter plots of the relationship between age and gender adjusted residuals of MMSE performance and extracted hippocampal GM values (i.e. TIV adjusted residuals; r- and p-values based on the primary partial correlation). White arrows indicate an outlier that was removed in additional analyses (see results section). L HC, left hippocampus; R HC, right hippocampus; MMSE, Mini Mental State Examination * p < 0.001 (two-sample *t*-test, two-tailed).

**Fig. 4:**
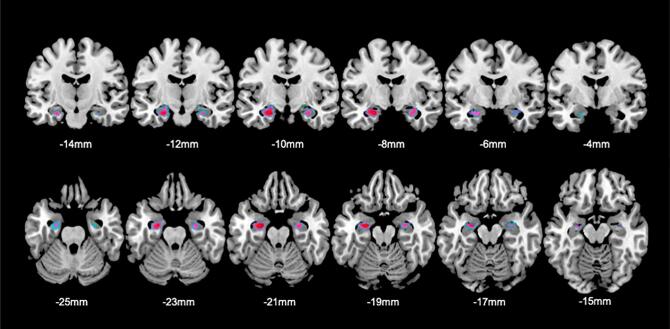
Greater regional GMV (VBM) in TGA + vs. TGA- after exclusion of 2 participants. Results of ROI-based VBM analyses after exclusion of N = 2 participants (TGA+: N = 19; TGA-: N = 19) with macrostructural hippocampal abnormalities upon neuroradiological examination. These results show comparable hippocampal GMV changes increases (p < 0.05 FEW_SVC_) in TGA + vs. TGA- as found in our main analyses with the entire sample (left HC: p < 0.01; k_E_ = 308; MNI_xyz_ [-24, −9, −20 mm]; right HC: p = 0.02; k_E_ = 245; MNI_xyz_ [30, −8, −18 mm]. Within the hippocampal ROI, both clusters most probable corresponded to the CA1 subfield according to the SPM Anatomy Toolbox. Coordinates indicate the corresponding location on the y-axis (top row) and z-axis (bottom row), respectively.

Whole brain analyses of regional GMV did not reveal statistically significant differences between TGA + and TGA- for both contrasts (i.e. “TGA+ < TGA”- and “TGA+ > TGA”-) within the hippocampal (HC) region. Yet, within the prefrontal cortex (i.e. BA6; see Fig. 2), TGA + showed lower GMV in comparison to TGA- (p = 0.037; k_E_ [TFCE-based cluster extent] = 31; MNI_xyz_ [-36, 10, 52 mm]). On prespecified ROI analysis of the bilateral hippocampus, we found significantly greater bilateral hippocampal GMV (left HC: p < 0.02; k_E_ = 219; MNI_xyz_ [−24, −8, −28 mm]; right HC: p = 0.04; k_E_ = 50; MNI_xyz_ [27, −9, −22 mm]; see Fig. 3A-C) in TGA + vs TGA-. Both clusters corresponded to the CA1 subfield according to the SPM Anatomy Toolbox. For the inverse contrast (i.e. TGA+ < TGA-), no significant differences were observed. A second ROI analysis after exclusion of the two participants with macrostructural abnormalities of the hippocampus upon neuroradiological inspection revealed a comparable result (Fig. 4). Additional effect size analyses (i.e. Cohen’s d) using the extracted GM data from the left and right HC (cluster average, adjusted for TIV) indicated a large effect size (i.e. 1.41 and 1.31for the left and right HC), supporting the robustness of our finding.

Across the entire population of N = 40 subjects, exploratory post-hoc analyses of extracted GM data from the left and right HC (cluster average, adjusted for TIV) showed a significant inverse correlation between left hippocampal GMV and global cognitive function as measured by the MMSE (see Fig. 3C, left HC: r = −0.34, p = 0.03, partial correlations adjusted for age and gender). For the right HC a similar inverse association was observed, yet at trend-level significance only (r = −0.27, p = 0.1; partial correlations adjusted for age and gender). However, no significant correlations (all p > 0.05) were observed when both groups were analyzed separately, (TGA+: left HC: r = −0.32; right HC: r = −0.27; TGA-: left HC: r = 0.09; right HC: r = 0.16). Fisher-Z-transformation did not yield significant differences of the respective correlations between TGA+ and TGA−, although a trend was observed (p = 0.11 for the correlation of MMSE with right HC and p = 0.10 for the correlation with left HC). Furthermore, exclusion of the one outlier (marked in Fig. 3C) strongly attenuated these results, leading to non-significant negative correlations only (i.e. left HC: r = −0.19, p = 0.26; right HC: r = −0.22, p = 0.17). In addition, there was no significant correlation of hippocampal GMV with any other cognitive measure (e.g. VVM-path length, CVLT, autobiographical memory and Human Water Maze performance). Also, no associations were observed between left or right hippocampal GMV and the time delay (i.e. date of study MRI minus date of TGA; age and gender adjusted partial correlation). Additional post-hoc analyses of extracted GM data from the left prefrontal cortex (cluster average) did not yield any significant associations with our measures of cognitive performance. Inclusion of educational level as additional covariate did not significantly change the results of these post-hoc analyses.

## 4 Discussion

This study investigated memory performance together with hippocampal gray matter volume (GMV) in the long-term course years after the TGA event. We found lower navigation performance in the huWMZ and in the MMSE total score in patients with (TGA+) compared to healthy controls without a history of TGA (TGA−), which may be indicative for subtle persisting hippocampal dysfunction. Contrary to our hypothesis, voxel-based morphometry (VBM) revealed greater GMV in the hippocampus bilaterally in TGA + compared to TGA-, which negatively correlated with the MMSE, i.e. greater hippocampal GMV was associated with lower scores in the MMSE. In the following, we will critically discuss these findings in the light of previous findings and methodological constraints.

Neurocognitive testing revealed slight, though significant impairments of cognitive performance in TGA+ as indexed by a small difference in the total score of the MMSE and longer path lengths in the huWMZ. However, in both measures, detailed analyses clearly speak against substantial long-term memory-related hippocampal impairment caused by the TGA. Regarding the MMSE, subanalyses of the retrieval task (delayed recall of the 3 words), as the major hippocampus-dependent part of the MMSE, no significant differences between the groups were shown. Furthermore, after the exclusion of one outlier in TGA+, difference between both groups was no longer significant. Similarly, in the huWMZ, increased path lengths were not accompanied by increased latencies. Since deficits in visuospatial learning/navigation are typically associated with an increase of both, path length and latency, substantial long-term impairment in visuospatial memory appears unlikely. Rather, the result suggests that TGA patients took little time for stops to identify context and to make decisions, a behavior, not directly attributable to hippocampal dysfunction. In contrast to our observations in the long-term course, in patients with acute TGA, increases in both, paths lengths and latencies have been demonstrated (Bartsch et al., 2010). In this work, a correlation between the size of the acute DWI-lesions and the severity of deficits in the visuospatial performance emphasized the critical role of hippocampal CA1 neurons to visuospatial learning and their involvement in the pathophysiological mechanisms during acute TGA. With respect to the other results of our neurocognitive testing, there was no evidence for a long-term selective disorder of episodic verbal or autobiographical memory years after the TGA. Since we used the short version of the CVLT, one may argue that possible ceiling effects might obscure subtle long-term impairments in episodic verbal memory in TGA patients. However, since for the immediate recall total score, mean subjects’ results were more than 1 standard deviation below the maximum score, this is unlikely. In sum, our results do not support substantial hippocampus-dependent deficits in the long-term course after TGA.

With regard to the VBM results, greater hippocampal GMV in TGA+ vs TGA- was unexpected and appears at odds with past research demonstrating that patients with TGA have significant GMV reduction in the hippocampus (Park et al., 2015) and widespread alterations in cortical morphology (including cortical thickness and volume) compared to healthy controls (Kim et al., 2017). In our study, differences in GMV were mainly located within the CA1 subfield of the hippocampus. Since the CA1 subfield is critically involved in the pathophysiology of TGA (Bartsch et al., 2011, Bartsch et al., 2015), spatial specificity of the VBM finding provides indirect support for the solidity of our finding. We consider it likely that the discrepancy between our findings and those obtained previously by other groups may be explained by different study designs, especially by different latencies between symptom onset and MRI scanning. In previous studies, MRI scans were performed during or shortly after the TGA episode. In contrast, we investigated brain structural alterations with a mean time interval of 6.5 years between TGA and structural MR imaging. At the cellular level, it has been postulated that cytotoxic edema caused by glutamate-mediated calcium influx mainly in neurons of the CA1 region underlies subacute DWI signals associated with TGA (Ogawa et al., 2018). Conceivably, cytotoxic edema in the hippocampus could be responsible for the observed volume change. However, cytotoxic edema as an acute or subacute phenomenon is unlikely to persist after 6,5 years. Possible alternative mechanisms underlying the macrostructural volume difference in TGA+ vs TGA- may include adult hippocampal neurogenesis (Kempermann et al., 2015). This is a complex and highly regulated multistep process in which new excitatory granule cells in the dentate gyrus are generated and sent to area CA3 along mossy fibres over an extended period of time (Gould et al., 1999). Hippocampal neurogenesis may have been triggered by pathophysiological mechanisms coming to effect during TGA and may have evolved to produce the long-term macroscopic changes in the hippocampus observable with MRI. However, GMV increases detectable with MRI have been noted in other circumstances in brain regions devoid of neuronal stem cells and hence incapable of neurogenesis. In these regions, non-neuronal components such as gliogenesis or vascular changes have been discussed as candidate mechanisms (Zatorre et al., 2012). Whatever precise mechanisms may underlie the GMV difference observed between our cohorts, our results clearly argue against TGA-induced atrophy as a long-term consequence of TGA. This conclusion is in line with a recent investigation of TGA patients using ultra high-field 7T MRI showing no structural abnormalities four months post TGA at the site of the previous DWI lesion (Paech et al., 2020).

When looking at the relationship of our VBM finding with the neurocognitive testing, we found greater GMV in the hippocampus to be associated with poorer performance in global cognition (MMSE). This relationship differs from observations of e.g. Maguire and colleagues (2000), who reported that in normal subjects, larger posterior hippocampal volume was related to greater experience-dependent visuospatial abilities. Of note, when excluding one outlier, the correlation coefficients remained negative but failed significance. In addition, none of our hippocampus-dependent measures correlated with hippocampal GMV. Therefore, at this point, we can only speculate, that mechanisms impacting the hippocampus after TGA are different from those triggered by extensive visuospatial training.

Finally, we would like to point to some limitations of our study. First, the cross-sectional study design hampers the interpretation of our VBM finding. The missing longitudinal time dimension makes it questionable to assume a change of hippocampal GMW over time. Alternatively, differences in GMV in the hippocampi could reflect a predisposition for TGA. In this way, patients with greater hippocampal GMV would be more likely to develop a TGA. Because of the retrospective selection of patients, we cannot test this alternative explanation directly. In addition, the rather small sample size limits the statistical power, particularly for robust relationships between structure and behavior.

In sum, patients with a history of TGA showed greater hippocampal GMV compared to controls in the long-term course after TGA. This finding was robust and supported by strong effect sizes bilaterally. Although we found no relationship between this finding and hippocampus-dependent performance, our results suggest GMV increase caused by (non-)neuronal components rather than atrophy. Longitudinal within-subject studies are needed to confirm our findings and to examine the nature of the relationship between changes in hippocampal GMV and cognitive sequelae.
